# Ageing, Sex Differences, and REDs Risk in Endurance Runners: An Integrated Cross-Sectional Study Protocol

**DOI:** 10.3390/sports14030121

**Published:** 2026-03-19

**Authors:** Ľudmila Oreská, Barbora Kundeková, Lukáš Varga, Katarína Stebelová, Monika Okuliarová, Juraj Payer, Milan Sedliak

**Affiliations:** 1 Department of Biological and Medical Sciences, Faculty of Physical Education and Sports, Comenius University in Bratislava, 814 69 Bratislava, Slovakia; 2 Department of Health Technologies, Faculty of Pharmacy, Comenius University in Bratislava, 832 32 Bratislava, Slovakia; 3 Department of Otorhinolaryngology Head and Neck Surgery, Faculty of Medicine and University Hospital Bratislava, Comenius University, 851 07 Bratislava, Slovakia; 4 Department of Animal Physiology and Ethology, Faculty of Natural Sciences, Comenius University in Bratislava, 842 15 Bratislava, Slovakia; 5 Department of Internal Medicine, Faculty of Medicine, Comenius University in Bratislava, University Hospital, 826 06 Bratislava, Slovakia

**Keywords:** ageing, master athletes, endurance running, sex differences, relative energy deficiency

## Abstract

Endurance performance is influenced by age- and sex-specific physiological determinants, while emerging evidence indicates an increasing prevalence of Relative Energy Deficiency in Sport (REDs) among both young and master endurance runners. Despite its clinical relevance, limited data exist on how long-term endurance training modulates REDs risk, skeletal muscle characteristics, and physiological ageing in comparison with inactive individuals. **Methods:** This cross-sectional study protocol will examine 112 participants stratified by sex, age (20–35 vs. 65–80 years), and training status (endurance runners vs. inactive controls). Cardiorespiratory fitness (VO_2_max) is defined as the primary outcome. Secondary outcomes include body composition, musculoskeletal function, biochemical and hormonal markers, and REDs-related screening variables. Assessments will comprise cardiorespiratory testing, DXA-based bone and body composition analysis, isometric knee dynamometry, mobility testing, validated REDs screening tools (LEAF-Q, LEAM-Q, and IOC REDs CAT2), seven-day dietary and training monitoring, venous blood sampling, and skeletal muscle biopsies from the vastus lateralis. **Results:** This study is designed to generate an integrated overview of physiological, nutritional, metabolic, and muscle-cell characteristics across sex-, age-, and training-specific subgroups. **Conclusions:** This protocol provides comprehensive insight into how ageing and sex influence endurance physiology and REDs susceptibility and whether long-term endurance training preserves functional capacity across the lifespan. The findings aim to support evidence-based screening, prevention, and targeted interventions for REDs in endurance athletes.

## 1. Introduction

Endurance running performance is shaped by a complex interplay between physiological, biomechanical, and sociocultural factors, with biological sex and ageing representing two of the most influential determinants across the lifespan. Across all levels of competition, male runners typically outperform female runners in events ranging from middle-distance to ultramarathons. This performance disparity is primarily attributed to sex-specific physiological characteristics, including higher maximal oxygen uptake (VO_2_max), greater cardiac stroke volume, elevated haemoglobin concentrations, and larger skeletal muscle mass in men, which collectively enhance oxygen transport and utilisation during prolonged exercise. These differences largely reflect divergent hormonal environments, with testosterone promoting muscle hypertrophy, erythropoiesis, and aerobic capacity, thereby supporting higher aerobic power and endurance performance in male athletes [1,2,3].

However, sex-based differences in endurance performance extend beyond aerobic capacity alone. Female runners exhibit sex-specific biomechanical and metabolic characteristics, including a greater reliance on lipid oxidation during submaximal exercise, a higher proportional area of type I muscle fibres, and pacing strategies that may be more even in prolonged events, which may contribute to performance in ultra-endurance contexts [4,5]. Despite these adaptations, males typically outperform females in endurance events, with the magnitude of the sex-based performance gap varying by discipline, event demands, and competitive level (approximately 10–30% in many athletic contexts) [2,4,5]. Endogenous hormonal fluctuations across the menstrual cycle may influence selected physiological responses to exercise in female athletes; however, the current evidence linking menstrual-cycle phase to performance outcomes remains mixed and inconclusive. Findings are highly variable between individuals, and results differ across study designs and methodological approaches [1,6]. Ageing introduces an additional layer of complexity to endurance performance. Master athletes experience a progressive decline in physiological capacity, primarily driven by reductions in VO_2_max, maximal heart rate, stroke volume, and skeletal muscle mass. After approximately 70 years of age, the rate of performance decline accelerates, exceeding 1.3% per year in both sexes. While older male runners generally retain higher absolute performance levels, they often exhibit steeper relative declines compared with female runners, suggesting that ageing may partially attenuate sex-based performance differences, particularly in long-duration endurance events [5,7,8].

Beyond performance-related determinants, endurance running is increasingly associated with the risk of Relative Energy Deficiency in Sport (REDs), a syndrome resulting from prolonged low energy availability and affecting multiple physiological systems, particularly endocrine, metabolic, musculoskeletal, and cardiovascular function. REDs has been documented in both male and female endurance runners across age groups, with sex-specific manifestations driven by hormonal and physiological variability. Female athletes tend to exhibit a greater number of affected physiological systems, with previous studies reporting an average of 2.9 affected systems in females (most frequently reproductive, endocrine, skeletal, and metabolic) compared with 1.6 in males (most frequently endocrine, skeletal and haematological) [9]. Furthermore, female runners more frequently report menstrual dysfunction, disordered eating behaviours, and compulsive training patterns, all of which are strongly linked to REDs development. Importantly, emerging evidence suggests that the prevalence of REDs may be increasing among ageing master endurance runners, potentially due to cumulative training loads, age-related changes in energy requirements, and inadequate nutritional compensation [10]. Although long-term endurance training may attenuate certain aspects of physiological ageing, insufficient energy intake relative to expenditure may exacerbate hormonal disturbances, impaired bone health, and maladaptive musculoskeletal changes. Targeted training strategies combined with evidence-based nutritional interventions, therefore, represent a critical component in mitigating REDs risk across the lifespan, not only in master athletes but also as a preventive strategy in younger endurance runners.

In this context, endurance training, energetic status, and endocrine milieu should be viewed as interacting determinants of ageing-related adaptation rather than as independent themes. While long-term endurance training may attenuate typical age-related declines in cardiorespiratory fitness and musculoskeletal function, these benefits likely depend on whether energy intake sufficiently supports the energetic cost of training, daily living, and recovery [11]. A sustained mismatch between intake and expenditure (i.e., problematic LEA exposure and/or chronic energetic stress) can contribute to the REDs spectrum through a multisystem endocrine–metabolic cascade, with downstream consequences for bone remodelling, skeletal muscle maintenance, substrate utilisation, recovery capacity, and ultimately performance [12,13,14]. Importantly, sex and reproductive life stage are expected to modify both susceptibility and clinical expression of this pathway (e.g., menstrual status vs. postmenopause), thereby influencing how energetic stress interacts with ageing processes and endurance-training adaptations in women versus men [12,13,14,15].

Consequently, a multidisciplinary approach integrating sports medicine, nutrition, and training science is essential for the early identification and management of REDs. Such an approach enables timely screening, individualised intervention, and improved long-term health and performance outcomes in endurance athletes of both sexes.

Therefore, the main aim of this study protocol is to comprehensively examine how biological sex and ageing influence physiological performance, skeletal muscle characteristics, bone health, and REDs risk in young and master endurance runners. The secondary aim is to compare endurance runners with their age- and sex-matched inactive counterparts to determine the extent to which long-term endurance training preserves physiological function and mitigates age-related decline across the lifespan. To address these aims, the primary outcome of this study is cardiorespiratory fitness, assessed as VO_2_max, while musculoskeletal, metabolic, endocrine, and REDs-related parameters are treated as secondary outcomes, providing complementary physiological context.

## 2. Materials and Methods

### 2.1. Study Design

**Figure 1:**
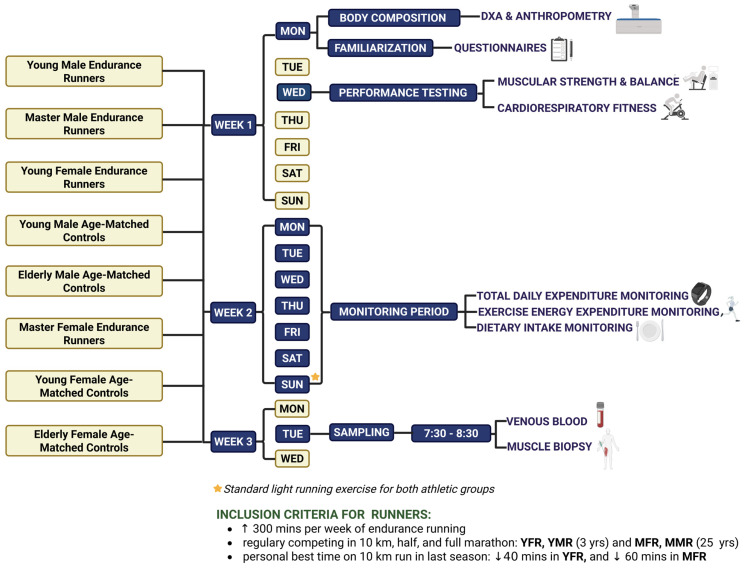
The cross-sectional study design. (Created with BioRender.com.)

The overall study design, illustrated in Figure 1, follows a cross-sectional approach. The study protocol was developed in accordance with the SPIRIT guidelines and is comprehensively documented using the SPIRIT checklist (Supplementary File S1), ensuring that all essential items required for reporting study protocols are systematically addressed.

### 2.2. Sample Size

Based on the planned between-group comparisons across eight study groups (sex × age category × training status), an a priori sample size calculation for an omnibus factorial ANOVA (fixed effects) was performed (α = 0.05, power = 0.90). Assuming a mean effect size of Cohen’s f ≈ 0.42, the required total sample size was *n* = 112, corresponding to 14 participants per group. The sample size estimation was performed using the G*Power software (version 3.1.9.2).

### 2.3. Study Subjects

A total of 112 subjects will be enrolled in this cross-sectional study and stratified into eight groups according to sex, age, and athletic status: (1) Young Male Endurance Runners, (2) Master Male Endurance Runners, (3) Young Male Age-Matched Controls, (4) Elderly Male Age-Matched Controls, (5) Young Female Endurance Runners, (6) Master Female Endurance Runners, (7) Young Female Age-Matched Controls, and (8) Elderly Female Age-Matched Controls. The young adult groups will include subjects aged 20–35 years, while the master and elderly groups will include individuals aged 65–80 years. Endurance runner groups will include elite-level Slovak athletes actively competing in national and international long-distance events. Young endurance runners will be defined as those with ≥3 years of national-level competitive experience, whereas master runners will be characterised by >5 years of continuous competitive participation. Group inclusion will be further validated using performance benchmarks of ≤35 min (young) and ≤55 min (masters) in a 10 km run. Control groups will comprise non-athletic individuals matched by sex and age, reporting <150 min of moderate and <75 of vigorous weekly activity during the past five years. This distinction reflects sustained physiological performance and adaptation in the context of ageing between both sexes [16].

The study will be conducted in accordance with the Declaration of Helsinki. All participants will receive detailed information about the study aims, procedures, and potential risks, and written informed consent will be obtained prior to enrolment. Ethical approval has been granted by the Ethical Committee of the University Hospital Bratislava—Hospital of Ladislav Dérer (No. 31/2020).

#### 2.3.1. Inclusion Criteria

Subjects will be considered eligible if they meet all the following criteria. Young adult groups will comprise individuals aged 20–35 years, whereas older adult groups will comprise individuals aged 65–80 years. Endurance-trained participants must perform ≥300 min·week^−1^ of structured endurance running, consistently maintained for at least three years, and compete at the national or international level. Physically inactive participants must report ≤150 min·week^−1^ of moderate-intensity activity and ≤75 min·week^−1^ of vigorous-intensity activity over the same period. Eligible participants must have a BMI between 18.5 and 35.0 kg·m^−2^ and sufficient Slovak language proficiency to understand study procedures and instructions (Supplementary File S2). All participants will provide written informed consent and will be required to comply with all study requirements.

#### 2.3.2. Female-Specific Considerations (Menstrual Status and Hormonal Contraception)

Hormonal contraception (HC) use will be recorded and classified (type, formulation, dose, and duration). Both naturally menstruating women and HC users will be eligible, provided that HC use has been stable for ≥3 months prior to enrolment.

To minimise heterogeneity, the primary analyses in female endurance runners will be performed in naturally menstruating participants, whereas HC users will be included in secondary (sensitivity) analyses and/or analysed as a separate subgroup if sample size allows. Where appropriate, HC status will be included as a covariate.

#### 2.3.3. Exclusion Criteria

Subjects will be excluded if they have musculoskeletal conditions that limit mobility or prevent participation in study assessments; acute or chronic infection; or diagnosed cardiovascular, neurological, metabolic, oncological, autoimmune, or other systemic diseases that contraindicate study involvement. Individuals with nutritional disorders (including malnutrition) or BMI < 18.5 kg·m^−2^ will also be excluded. The use of medications or substances within the past six months that may interfere with biological analyses (e.g., systemic corticosteroids, immunosuppressants, chemotherapeutic agents, hormonal treatments, or other immunomodulatory drugs) will constitute an exclusion criterion. In addition, the use of non-steroidal anti-inflammatory drugs (NSAIDs) within 24 h prior to any study visit will not be permitted and will result in rescheduling or exclusion, depending on frequency and clinical necessity. Any current or prior use of doping or performance-enhancing substances, major surgery or hospitalisation within the previous six months, and pregnancy or lactation will also result in exclusion (Supplementary File S2).

#### 2.3.4. Female-Specific Exclusions (Menstrual Status and Hormonal Contraception)

Young adult female subjects will be excluded if they have initiated, discontinued, or changed the type, dose, or delivery method of hormonal contraception within the past 3 months or if the contraceptive formulation and delivery method cannot be reliably documented (where applicable).

### 2.4. Familiarisation and Pre-Participation Medical Screening

Prior to enrolment, all prospective participants will complete a familiarisation session at the testing facility to ensure full understanding of study procedures, equipment, and the laboratory environment. This step is implemented to reduce procedural anxiety, improve compliance, and minimise learning effects during subsequent assessments.

Following familiarisation, a licensed sports medicine physician will conduct a comprehensive pre-participation medical evaluation, including medical history, resting heart rate and blood pressure measurements, physical examination, and resting and graded-exercise electrocardiography. The visit will also include a graded incremental cycling test under 12-lead ECG monitoring, which will serve both for clinical safety screening and for VO_2_max estimation (primary outcome). Additional assessments, such as spirometry, will be performed when clinically indicated.

Only participants who are medically cleared and present no contraindications to maximal or submaximal exertion will proceed to the study [17,18].

### 2.5. Primary Outcomes

Cardiorespiratory fitness expressed as estimated (VO_2_max) is defined as the primary outcome of interest, with secondary outcomes encompassing body composition, musculoskeletal function, biochemical and hormonal markers, and REDs-related screening variables. While VO_2_max (mL·kg^−1^·min^−1^) will serve as the primary clinical outcome, estimated VO_2_max normalised to fat-free mass (FFM) will be analysed as a secondary, mechanistically informative indicator of aerobic capacity relative to metabolically active tissue.

#### Cardiorespiratory Fitness Assessment

Maximal oxygen uptake (VO_2_max) will be estimated indirectly from the maximal power output (Wmax) achieved during a continuous incremental cycling test using the American College of Sports Medicine (ACSM) metabolic equation for leg cycling [18]:

$$VO_{2}max\;(mL\cdot kg^{-1}\cdot min^{-1})=\frac{11.016\cdot W_{max}}{BM}+7$$

where Wmax represents the maximal achieved power output in watts, and BM denotes body mass in kilograms. This equation is derived from the standard ACSM relationship between work rate and oxygen uptake during cycle ergometry and will be applied to estimate VO_2_max at peak exercise intensity. VO_2_max will serve as the primary physiological outcome, representing a validated indicator of aerobic capacity and cardiorespiratory health [19,20]. All testing will be conducted at the SPORTMED Sports Medicine Centre under medical supervision and in accordance with ACSM Guidelines for Exercise Testing and Prescription [18]. The test will begin with a 1 min rest and a warm-up at 20 W, followed by a continuous ramp protocol with workload increments of 20 W/min until volitional exhaustion or clinical termination. Increment size may be adjusted slightly (e.g., 25–30 W/min) based on sex, age, and predicted capacity to achieve an optimal test duration of 8–12 min, consistent with validated ramp protocols [21].

Heart rate and blood pressure will be monitored continuously via 12-lead ECG (Quark T12, Cosmed, Rome, Italy), and brachial blood pressure will be assessed before, during, and after exercise (Metronik BL-6, STOLL Medizintechnik, Germany). Maximum workload (in W and W·kg^−1^) will also be recorded. Tests will be terminated upon volitional fatigue, abnormal ECG responses, or safety-related criteria, following ACSM termination guidelines [18].

Secondly, to account for differences in body composition between sexes and age groups, VO_2_max will be normalised to fat-free mass obtained by dual-energy X-ray absorptiometry (DXA) by using the following equation:

$$VO_{2}max\text{\_}FFM\;(mL\cdot kg^{-1}FFM\cdot min^{-1})=\frac{VO_{2}max\text{\_}abs}{2aFFM}$$

All assessments will be administered by a licensed sports physician and a trained exercise physiologist to ensure participant safety, standardisation, and data quality.

### 2.6. Secondary Outcomes

#### 2.6.1. Anthropometric and Body Composition Assessment

Basic anthropometric variables, including standing height and body weight, will be collected as input parameters for subsequent body composition analyses. Standing height will be measured to the nearest 0.1 cm using a digital stadiometer (BSM 170, InBody Co., Ltd., Cerritos, CA, USA), with participants barefoot and positioned upright. Body weight will be assessed to the nearest 0.1 kg using a calibrated bioelectrical impedance analyser (InBody 230, InBody Co., Ltd., Cerritos, CA, USA), with subjects wearing minimal clothing.

The bioimpedance system (InBody 230, InBody Co., Ltd., Cerritos, CA, USA) will be used to obtain standardised body weight and descriptive estimates of body fat percentage and skeletal muscle mass for the calculation of strength normalised to body mass and/or skeletal muscle mass, whereas DXA will serve as the reference method for detailed regional body composition, fat-free mass (FFM), and all bone-related outcomes [22,23]. Therefore, to obtain a comprehensive and accurate assessment of body composition, all participants will undergo whole-body Dual-Energy X-ray Absorptiometry (DXA). Scans will be performed using standardised positioning procedures in the supine position, with careful alignment of the head, trunk, and limbs to minimise movement artefacts. All assessments will be conducted in the radiology department under the supervision of a certified radiologist.

DXA will provide a full set of bone-related parameters, including bone mineral content (BMC; g), bone mineral density (BMD; g·cm^−2^), and T-scores for whole body, proximal femur, and lumbar spine, enabling evaluation of bone health status. Additionally, body composition outcomes will be obtained, including total and regional lean soft tissue mass, fat mass, body fat percentage, appendicular lean mass (ALM), and indices relevant for functional and clinical assessment, such as the appendicular lean mass index (ALMI; kg·m^−2^) and fat-free mass index (FFMI). Regional compartmental analysis (arms, legs, trunk) will also be performed. Furthermore, in accordance with the recent viewpoint advocating for sport-specific bone mineral density reference values for athletes rather than general population thresholds, we will compare our runners’ bone health classifications using both conventional clinical criteria and the updated athlete-oriented cut-offs [24].

#### 2.6.2. Assessment of Lower Body Strength

Lower-body strength will be assessed using an isometric knee dynamometer (ARS dynamometry, S2P Ltd., Ljubljana, Slovenia) to determine maximal voluntary contraction (MVC) of the knee extensors and flexors, following validated procedures [25,26]. Testing will be performed in a custom-designed isometric chair adjusted individually to ensure optimal joint alignment and reproducibility, in accordance with established dynamometry guidelines [27,28].

Prior to MVC assessment, subjects will complete two submaximal warm-up contractions at approximately 50% and 80% of perceived maximal effort, separated by 30 s of rest, to minimise variability and enhance measurement accuracy [28]. Subjects will then perform three maximal isometric trials for both knee extension and flexion. Each contraction will involve a rapid force production sustained for five seconds, with strong verbal encouragement provided. A 90 s rest will be given between trials, and a 3 min rest will be given between muscle groups to limit fatigue and potentiation effects [29,30].

Peak torque and the rate of torque development (RTD) will be recorded at 0–50 ms, 0–100 ms, 0–150 ms, and 0–200 ms to capture early-phase neuromuscular performance relevant for athletic capacity and age-related functional decline [31,32]. The highest-performing trial will be retained for analysis. To normalise relative MVC, the following formulas will be used to enable better interindividual comparisons among groups:

(1) peak torque (PT; Nm) to body mass (m; kg):

$$RelativePT\left(Nm\cdot kg^{-1}\right)=\frac{PT\left(Nm\right)}{m\left(kg\right)}$$

(2) peak torque (PT; Nm) to total lean body mass (LBM; kg):

$$RelativePT\left(Nm\cdot k{g\;LBM}^{-1}\right)=\frac{PT\left(Nm\right)}{LBM\left(kg\right)}$$

(3) peak torque (PT; Nm) to thigh lean mass from DXA segment analysis (thigh LBM; kg):

$$RelativePT\left(Nm\cdot k{g\;thigh\;LBM}^{-1}\right)=\frac{PT\left(Nm\right)}{thigh\;LBM\left(kg\right)}$$

### 2.7. Mobility

#### 2.7.1. Y-Balance Test

Dynamic balance will be evaluated using the Y-Balance Test (YBT) [33,34]. Subjects will perform the test barefoot, completing one familiarisation trial per limb to minimise learning effects, followed by one recorded trial for each leg.

During testing, subjects will maintain a single-leg stance on the central footplate while reaching maximally with the contralateral limb in the anterior, posteromedial, and posterolateral directions. Balance must be maintained without shifting the stance foot or using the reaching foot for support. Reach distances will be measured in centimetres and normalised to limb length, assessed from the anterior superior iliac spine to the distal medial malleolus [34].

#### 2.7.2. Dietary Intake Monitoring and Nutritional Analysis

Following the familiarisation session, subjects will be instructed to maintain their habitual dietary patterns during the seven-day monitoring period. They will receive standardised instructions for completing dietary records, including estimation of portion sizes and optional photographic documentation to enhance accuracy [35]. Dietary intake will be recorded for seven consecutive days (Monday–Sunday) using structured food logs.

A certified nutrition specialist will analyse all records using the Planeat 365 software (Planeat s.r.o., Bratislava, Slovakia). The analysis will include total daily energy intake, absolute and average daily macronutrient intake (carbohydrates, proteins, fats), and the temporal distribution of nutrient intake to identify within-day energy availability patterns [36,37].

#### 2.7.3. Seven-Day Training Monitoring Period

All endurance running groups of subjects will maintain a 7-day training diary throughout the monitoring period. For each training session, subjects will record exercise modality, session duration, distance covered, running pace, elevation gain, perceived intensity, and training frequency. Perceived intensity will be quantified using the session rating of perceived exertion (sRPE) method: subjects will report an overall session RPE on the modified Borg CR-10 scale (0–10) approximately 30 min after each session, following standardised written instructions and verbal anchoring. Participants will be familiarised with the CR-10 scale before the monitoring period using example sessions to ensure consistent interpretation [38]. Internal training load will be calculated as sRPE training load (TL) = session RPE × session duration (min) and summarised as daily and 7-day cumulative training load [39].

To enhance data accuracy and completeness, training records will be complemented by session data exported from Strava (Strava Inc., San Francisco, CA, USA), including time-stamped activity files and external workload parameters. Strava-derived metrics (duration, distance, pace, and elevation gain) will be used to characterise external training load and to cross-check diary entries, whereas sRPE-derived load will be used to capture internal load. These combined records will be used to characterise individual training load and to support the estimation of exercise energy expenditure.

To further characterise habitual physical activity patterns beyond structured training, daily movement behaviour will be objectively monitored using a wrist-worn triaxial accelerometer (MotionWatch 8 ©, Cambridge Neurotechnology, Fenstanton, Cambridgeshire, UK). Participants will wear the device continuously for seven consecutive days, concurrent with the training diary period. Downloaded actigraphy data will be analysed using the MotionWare software (MotionWare 1.2.31, Cambridge Neurotechnology, Fenstanton, Cambridgeshire, UK) to derive daily wear time, to identify valid monitoring days, and to generate Day Activity Analysis summaries, including counts and duration of activity across intensity thresholds. Time spent in sedentary, light, moderate, and vigorous intensity activity will be quantified (min·day^−1^) based on the activity count thresholds configured in MotionWare, and moderate-to-vigorous physical activity (MVPA) will be calculated as the combined duration of moderate and vigorous activity. In addition, average daily activity counts will be extracted from MotionWare outputs as an index of overall movement volume independent of intensity categorisation. Given the wrist-based placement of the accelerometer, participants will be explicitly instructed not to cover the device with clothing or accessories during the monitoring period, either indoors or outdoors, to minimise signal attenuation and ensure consistent detection of movement-related activity. These parameters will be used to complement the training diary and Strava-derived data, allowing for a comprehensive assessment of daily physical activity exposure [40,41].

### 2.8. Exploratory Outcomes

#### 2.8.1. Blood Sample Collection, Biochemical and Metabolic Analyses

The performance-testing visit will be followed by a 7-day free-living monitoring period (diet and activity). Blood sampling and muscle biopsy will be scheduled within the subsequent week, after at least 48 h without strenuous exercise, to minimise acute exercise-induced biomarker perturbations. Overall, sampling will occur approximately 10–14 days after performance testing, depending on participant availability and monitoring completeness. Upon completion of monitoring, participants will observe a short period without structured training to minimise acute exercise-induced alterations in biochemical, metabolic, and endocrine markers. Blood sampling will, therefore, reflect habitual physiological status rather than short-term post-exercise responses.

To minimise acute exercise-related fluctuations in circulating biomarkers, participants will refrain from strenuous activity for at least 48 h prior to sampling [42,43,44,45]. During this period, young and master endurance runners will be allowed one low-intensity training session in heart rate zone 2 (60–70% HRmax), limited to 60 min for young adults and 45 min for masters, to maintain habitual physiological states without provoking acute metabolic or endocrine responses [46].

Blood sampling will be performed by a certified nurse at the hospital between 7:00 and 7:30 a.m. Subjects will arrive by private transport, rested, and in an overnight fasted state (≥10 h) to standardise hormonal and metabolic conditions across groups.

Biochemical, metabolic, and endocrine analyses will be conducted in ISO-accredited commercial laboratories. The assay panel will include a comprehensive panel of haematological, biochemical, metabolic, inflammatory, and hormonal markers, as well as markers of energy availability, bone metabolism, and endocrine status, with particular attention to biomarkers relevant to the Female Athlete Triad and Relative Energy Deficiency in Sport (REDs) [47,48].

Hormonal status will be evaluated through measurement of sex hormones and related indices, including estradiol, total testosterone, free testosterone, biologically available testosterone, sex hormone-binding globulin (SHBG), and derived androgen indices. In female participants, menopausal status will be recorded (see Menopausal Status section). Hormonal data will be interpreted in relation to sex, age category, and training status in order to control for potential confounding effects of hormonal variability and to support physiologically meaningful interpretation of endocrine outcomes.

Insulin resistance as a metabolic parameter will be estimated using the Homeostatic Model Assessment of Insulin Resistance (HOMA-IR), calculated as

*HOMA-IR* = [*Fasting Insulin* (μU/mL) × *Fasting Glucose* (mmol/L)]/22.5

#### 2.8.2. Menopausal Status

In female participants, menopausal status will be assessed using a structured questionnaire, including the age at natural menopause onset and current bleeding status. Self-reported menopausal status will be contextualised using circulating sex hormone concentrations obtained from venous blood analyses to provide objective endocrine support for age-related hormonal status. Menopausal status will be recorded and reported as a descriptive participant characteristic and considered in the interpretation of hormonal, metabolic, and bone-related outcomes, without being used as a diagnostic or primary grouping criterion.

#### 2.8.3. Muscle Biopsy

Muscle biopsies will be obtained at 08:00 a.m. following fasting blood collection. Muscle biopsy procedures are clearly described in the informed consent provided prior to enrolment, and participants will be informed that participation in this procedure is voluntary. Biopsies will be performed by a licensed physician with nursing assistance at the hospital. Under local anaesthesia (2% lidocaine), a percutaneous biopsy will be taken from the mid-portion of the right vastus lateralis using a 5 mm Bergström needle with manual suction to optimise tissue yield [49], followed by standardised processing procedures for molecular and immunohistological analyses [45].

Post-collection, visible connective and adipose tissue will be removed. A sample for immunohistochemistry will be embedded in OCT, snap-frozen in isopentane cooled in liquid nitrogen for cryosectioning. The remainder will be snap-frozen in liquid nitrogen. Both types of samples will be stored at −80 °C until further analysis [45]. For transmission electron microscopy (EM), the muscle samples will be fixed in freshly prepared 3.5% glutaraldehyde in 0.1 M cacodylate (NaCaCO) buffer, pH 7, and stored at 4 °C until embedding [8].

#### 2.8.4. Immunohistochemical Analysis of Skeletal Muscle Tissue

Frozen OCT-embedded muscle samples will be cryosectioned into 8 µm transverse sections at −20 °C using a calibrated cryostat (CM3050 S; Leica Microsystems, Wetzlar, Germany). Sections will be mounted on adhesive slides (Superfrost Plus; Thermo Fisher Scientific, Waltham, MA, USA), air-dried, and stored at −80 °C until immunohistochemical analysis.

Immunohistochemistry will be performed on all biopsy samples to quantify cellular and structural characteristics that will be used analytically, primarily as mechanistic markers of skeletal-muscle ageing and endurance-training adaptation across the sex × age × training-status groups. Specifically, we will assess myonuclear content, Pax7+ satellite cells (as indices of regenerative/remodelling capacity), capillary density using endothelial markers (e.g., CD31), and fibre-type distribution and fibre cross-sectional area using myosin heavy chain isoforms [50,51,52]. Group comparisons of these variables will address ageing- and training-related differences in muscle phenotype, whereas any links to REDs/LEA will be evaluated only in predefined exploratory association analyses (e.g., relating muscle phenotype to energetic/metabolic context such as energy intake/energy balance/EA estimates and complementary biopsy-derived metabolic measures). Image-based quantification will be conducted using ImageJ (Fiji)and the TEMA 95 software (Scanbeam A/S, Hadsund, Denmark).

#### 2.8.5. Electron Microscopy

Following fixation and embedding, quantitative ultrastructural analyses will be performed to assess calcium release units (CRUs; triads), mitochondria, and calcium entry units (CEUs). The following parameters will be quantified: (1) CRU density (number of CRUs per unit area); (2) mitochondrial density and the number of mitochondria–CRU associations per unit area [37,53]; and (3) CEUs, defined as specialised junctions formed by sarcoplasmic reticulum (SR) stacks and elongated transverse (T)-tubules at the I band. CEUs will be quantified by determining (a) the percentage of fibres containing SR stacks and the number of SR stacks per 100 μm^2^ and (b) both (i) the extent of SR closely associated with T-tubules per 100 μm^2^ and (ii) the total extension of the T-tubule network per 100 μm^2^ of muscle section. Electron-microscopy-derived parameters (CRU/triad density, mitochondrial density and mitochondria–CRU associations, and CEU-related indices) will be interpreted as mechanistic markers of muscle structure and remodelling capacity that are sensitive to ageing and long-term training exposure [8].

#### 2.8.6. Protein Expression

Protein expressions will be analysed by Western blotting using a standardised procedure adapted from previously published methods. Briefly, freeze-dried muscle tissue (~3 mg total; prepared as two ~1.5 mg portions) will be cleaned of visible blood, fat, and connective tissue and then homogenised. Total protein concentration will be measured in triplicate using a BCA assay (Thermo Fisher Scientific, Waltham, MA, USA). The same amount of protein will be loaded into each lane of a precast 16.5% gel (26 wells; Bio-Rad), with all samples from a given participant run on the same gel. Proteins will be detected by enhanced chemiluminescence (ECL; Merck Millipore, Merck KGaA, Darmstadt, Germany) and imaged using a ChemiDoc MP system (Bio-Rad Laboratories, Inc., Hercules, CA, USA). Band intensities will be quantified in Image Lab (v6.0; Bio-Rad) after background correction. OXPHOS proteins will be detected using Abcam ab110411 (1:1000) [54,55].

#### 2.8.7. Gene Expression (qPCR)

Muscle tissue allocated for molecular analyses will be snap-frozen in liquid nitrogen and stored at −80 °C until analysis. Total RNA will be isolated using TRI Reagent (Molecular Research Center, Cincinnati, OH, USA) according to the manufacturer’s instructions. RNA quantity and purity will be assessed using a NanoDrop One spectrophotometer (Thermo Fisher Scientific), and RNA integrity will be evaluated using a 2100 Bioanalyzer (Agilent Technologies, Inc., Santa Clara, CA, USA). Complementary DNA will be synthesised using the Maxima cDNA Synthesis Kit (Thermo Fisher Scientific, USA). Quantitative PCR will be performed using Maxima SYBR Green qPCR Master Mix (Thermo Fisher Scientific) on a CFX Connect real-time PCR detection system (Bio-Rad, USA). Target genes will include inflammatory markers (e.g., IL-1β, IL-6, TNFα, COX-2) and markers of muscle differentiation and atrophy (e.g., Atrogin-1, MuRF-1, FoxO, MyoD) [56].

#### 2.8.8. Skeletal Muscle Glycogen Content

Muscle glycogen content will be determined in freeze-dried muscle samples using a modified spectrophotometric approach based on the acid-hydrolysis method [57] and consistent with recent applications in human muscle biopsy studies [55,58]. Briefly, ~1–2 mg dry weight (d.w.) of muscle tissue will be extracted in 0.5 mL of 1 M HCl and hydrolysed at 100 °C for 3 h. Following hydrolysis, glucose will be quantified using the hexokinase method with a commercial glucose kit and a PentraC 400 analyser (TrioLab). NADPH formation will be measured spectrophotometrically at 340 nm and used to calculate glucose content, which is directly proportional to muscle glycogen concentration [57]. In addition, muscle pH will be assessed using a micro-glass electrode (XC 161, Radiometer-Analytical, Lyon, France) after homogenising ~1 mg d.w. of tissue in 100 μL of a non-buffered solution containing 145 mM KCl, 10 mM NaCl, and 5 mM sodium fluoride [59].

Gene-expression markers (inflammatory and atrophy/differentiation-related genes), Western blot targets (e.g., OXPHOS), and glycogen content will be treated as complementary molecular and metabolic descriptors of muscle phenotype. These measures are not intended as direct diagnostic indicators of REDs. However, predefined exploratory analyses will examine whether muscle phenotypes covary with the energetic/endocrine context relevant to LEA (e.g., energy intake/energy balance/energy availability estimates and selected hormonal and bone-health markers), with any relationships interpreted as hypothesis-generating.

### 2.9. Screening Outcomes (REDS/LEA Screening and Symptom-Based Risk Stratification)

Energetic status variables (mean eEB and exploratory EA) and REDs screening outputs (LEAF-Q, LEAM-Q, IOC REDs CAT2) will be analysed as exploratory, hypothesis-generating indicators. We will prioritise inference based on convergent patterns across domains (energetic metrics, screening tools, and objective physiological outcomes) rather than relying on any single threshold-based classification.

#### 2.9.1. Screening of Low Energy Availability: LEAM and LEAF Questionnaires

Risk of low energy availability (LEA) will be screened during the familiarisation session using sex-specific, validated questionnaires designed to detect early indicators of Relative Energy Deficiency in Sport (REDs). Male participants will complete the Low Energy Availability in Males Questionnaire (LEAM-Q), whereas female participants will complete the Low Energy Availability in Females Questionnaire (LEAF-Q). Each original version of the LEAF-Q and LEAM-Q has already been translated into the Slovak language according to the WHO [60].

The LEAM-Q targets male-specific physiological and behavioural domains, including training load, dietary habits, gastrointestinal symptoms, libido, injury history, and eating-/body-image-related attitudes [61]. It demonstrates acceptable reliability (Cronbach’s α = 0.83) and preliminary construct validity, particularly in relation to testosterone concentrations and markers of bone turnover [62]. Screening for LEA in males is important because REDs in men is often subclinical and may present with less overt symptoms than in females [46].

LEAM-Q consists of the following variables: dizziness, gastrointestinal function, resting thermoregulation, injury and illness interfering with training and competition, fatigue, fitness, sleep, recovery, energy levels, and sex [60]. Each variable will provide an individual score. Scoring of LEAM-Q is currently limited only to four questions related to sex drive and morning erections. Scores for LEAM-Q items will be calculated according to the published scoring approach, where higher scores for each participant will indicate a worse or more negative outcome of REDs incidence [63]. Because validated threshold-based scoring is currently limited for selected items, we will report total/summary scores where applicable and key item-level indicators (e.g., libido/morning erections) descriptively and interpret them alongside hormonal and bone-health markers.

The LEAF-Q is validated for females and screens LEA-related dysfunction across three key domains: menstrual function, gastrointestinal symptoms, and injury history [64]. It shows high sensitivity (78%) and specificity (90%), excellent test–retest reliability (r = 0.92), and good internal consistency (Cronbach’s α = 0.71). Chronic LEA in female athletes is associated with menstrual dysfunction, impaired bone health, and increased injury risk [47,48].

LEAF-Q contains 25 questions arranged into three variables: injury, gastrointestinal function and reproductive function. Questions are scored according to a key provided with the questionnaire, in which a total score of ≥8 out of 25 questions will serve as an indicator that female runners are at risk of LEA. Each variable will give an individual score.

Together, the LEAM-Q and LEAF-Q enable sex-specific assessment of LEA risk. Questionnaire scores will be compared between endurance athletes and age-matched inactive counterparts and examined in relation to hormonal, metabolic, physiological, and bone-health markers, providing an integrated evaluation of REDs-related outcomes [64].

#### 2.9.2. Menstrual Function

As part of the LEAF-Q, menstrual function will be assessed using items on cycle regularity and self-reported menstrual disturbances. Menstrual status will be classified using LEAF-Q-consistent criteria and standard clinical definitions of menstrual dysfunction [64]. Amenorrhoea will be defined as primary (no menarche by age 15 years) or secondary (absence of menses for >90 days), whereas eumenorrhoea will be defined as regular cycles occurring at ~21–35-day intervals. Participants will be categorised as eumenorrhoeic (regular cycles with recent bleeding within ~0–4 weeks) or as having menstrual disturbances (oligomenorrhoea/irregular cycles and/or amenorrhoea); these categories may be collapsed into normally menstruating (eumenorrhoea + regular) and menstrual disturbance (oligomenorrhoea and/or amenorrhoea and/or irregular). For young adult runners who regularly menstruate and do not use hormonal contraception, the menstrual cycle phase during testing will be recorded [65]. Participants reporting current hormonal contraception will be excluded from menstrual-function analyses because exogenous hormones may mask cycle-based outcomes. Postmenopausal master female runners (≥12 months without menses) will not complete cycle-based LEAF-Q menstrual items; menopausal status will be recorded and menstrual-cycle classifications treated as not applicable in this subgroup [64,66,67].

#### 2.9.3. The IOC REDs CAT2 Clinical Assessment Tool

To stratify the severity/risk of LEA and REDs development in endurance running groups, we will implement a baseline assessment of the 2023 IOC REDs CAT2 [14] Consensus Statement, replacing the original REDs CAT [68]. IOC REDs CAT2 is a three-step model, integrating (1) validated screening questionnaires or clinical interviews, (2) a severity or risk assessment based on the accumulation of primary and secondary indicators (e.g., biomarkers, BMD, and injury history), and (3) expert clinical evaluation and management where indicated [14]. Based on the presence of primary and secondary indicators, CAT2 will assign a participant a traffic light (green, yellow, orange, or red), where green colour represents no REDs or very low risk; yellow colour represents low to moderate risk; orange colour represents moderate to high risk; and red colour represents high risk of REDs development [69]. Traffic-light classification will be assigned using the validated IOC REDs CAT2 Severity/Risk Stratification Calculator (Scoring Tool 2), which operationalises the CAT2 indicator-weighting framework to support consistent scoring and category allocation [69,70]. Data collection will include a self-report online survey, blood work, and DXA measurement (BMD) within 21 days (DXA 30 days) of the cross-sectional testing.

#### 2.9.4. Energetic Status, Estimated Energy Balance, and LEA-Related Screening

In this study, energetic status will be characterised using a multimodal framework integrating dietary intake, objective activity monitoring, training logs, REDs screening questionnaires, clinical stratification, and physiological markers. Estimated energy balance (eEB) will be prioritised as the primary descriptive energy metric under free-living conditions, as accurate quantification of exercise energy expenditure required for “classical” energy availability (EA) is challenging in endurance runners. Accordingly, both eEB and EA will be treated as exploratory screening indicators and will not be used as stand-alone criteria for diagnosing REDs/LEA.

#### 2.9.5. Energy Intake and Components of Estimated Total Energy Expenditure

Energy intake (EI) will be assessed using 7-day participant-completed food diaries. Daily body mass (BM, kg) will be recorded, and fat-free mass (FFM, kg) will be obtained from dual-energy X-ray absorptiometry (DXA).

Habitual movement behaviour will be monitored using wrist-worn actigraphy (MotionWatch 8). Actigraphy data will be processed in MotionWare to identify valid monitoring days and extract daily time spent in sedentary, light, moderate, and vigorous intensity activity (t_sed, t_low, t_mod, t_vig; min·day^−1^) using predefined count thresholds. For energetic calculations, total accumulated time across all epochs will be used.

Resting metabolic rate (RMR) will be predicted using the Cunningham equation:

$$RMR_{day}=500+22\times FFM$$

Physical activity energy expenditure (PAEE) will be estimated from actigraphy-derived time spent in light, moderate, and vigorous intensity activity by assigning standard metabolic equivalent values (light: 2.0 METs; moderate: 4.0 METs; vigorous: 8.0 METs) and subtracting 1 MET to account for resting expenditure:

$$PAEE_{day}=BM\times [(2.0-1)\frac{t_{low}}{60}+(4.0-1)\frac{t_{mod}}{60}+(8.0-1)\frac{t_{vig}}{60}]$$

The thermic effect of food (TEF) will be estimated as 10% of daily energy intake:

$$TEF_{day}=0.10\times EI_{day}$$

Estimated total daily energy expenditure (eTEE) will be calculated as

$$eTEE_{day}=RMR_{day}+PAEE_{day}+TEF_{day}$$

Daily estimated energy balance (EB) will be calculated as

$$eEB_{day}=EI_{day}-TEE_{day}$$

and further normalised to fat-free mass (FFM) obtained from DXA as

$$EB_{F}FM,day=\frac{EI_{day}-TEE_{day}}{FFM}$$

expressed in kcal·kg FFM^−1^·day^−1^.

Mean eEB across the 7-day monitoring period will be summarised as a continuous variable and used as the primary energy-related screening indicator of sustained energetic deficit/surplus in free-living conditions.

#### 2.9.6. Exploratory Energy Availability Estimation

To enable comparison with the established LEA literature, EA will be calculated only as an exploratory estimate when exercise energy expenditure (EEE) data are sufficiently complete. EEE will be derived from the 7-day training diary and Strava-exported training files (Strava Inc., USA), capturing exercise modality, session duration, distance/pace, and perceived intensity. These records will be used to identify exercise bouts and estimate daily EEE. Daily EA will be calculated as

$$EA_{\mathrm{day}}=\frac{EI_{\mathrm{day}}-EEE_{\mathrm{day}}}{FFM}$$

and will be expressed as kcal·kg FFM^−1^·day^−1^.

Mean daily EA across the 7-day monitoring period will be reported descriptively. For contextual comparison with the established EA literature, exploratory EA values will also be summarised using commonly cited interpretive bands derived from controlled studies (adequate: ~45 kcal·kg FFM^−1^·day^−1^; reduced: 30–45 kcal·kg FFM^−1^·day^−1^; low: <30 kcal·kg FFM^−1^·day^−1^). These bands (including the ~30 kcal·kg FFM^−1^·day^−1^ threshold) will be used for contextual interpretation only and will not be applied as diagnostic cut-offs in free-living conditions, where uncertainty in EEE estimation and error propagation can be substantial [53,71]. Interpretation will follow the International Olympic Committee (IOC) consensus framework [31,35]. Calculated energetic metrics will be interpreted in conjunction with validated screening tools (LEAF-Q, LEAM-Q, and IOC REDs CAT2), which will be used as screening instruments within the REDs risk framework [14,46].

#### 2.9.7. Integration with REDs Screening Tools and Interpretation of Conflicting Indicators

REDs risk will be interpreted using a triangulation approach across multiple indicators, combining (i) questionnaire-based screening (LEAF-Q and LEAM-Q), (ii) energetic screening metrics (mean eEB and exploratory EA), and (iii) IOC REDs CAT2 clinical severity/risk stratification, in accordance with IOC guidance. Questionnaire scores and energetic metrics will be treated as screening indicators that support interpretation of REDs risk categories, while objective endocrine, metabolic, and bone outcomes will provide complementary physiological context. These tools will not be used diagnostically but rather as screening instruments within the REDs risk framework [14,46].

When indicators are discordant (e.g., a markedly negative mean eEB with low questionnaire-based risk or elevated questionnaire risk with near-neutral eEB), the results will be interpreted cautiously by considering potential sources of measurement/reporting error and by prioritising convergence with objective physiological markers and the overall clinical stratification framework rather than relying on any single metric [14,46].

#### 2.9.8. Statistical Analysis

Statistical analyses will be performed using standard statistical software (version 10; GraphPad Software, San Diego, CA, USA). Data will be inspected for completeness, normality, and homogeneity of variance prior to analysis. Normality of distribution will be assessed using the Shapiro–Wilk test, and homogeneity of variances will be evaluated using Levene’s test. Descriptive statistics will be reported as mean ± standard deviation (SD) for normally distributed variables or median (interquartile range) for non-normally distributed variables.

Given the breadth of the multimodal assessment, outcomes will be prioritised a priori to support interpretable inference. Cardiorespiratory fitness (estimated VO_2_max) will serve as the primary outcome. Secondary outcomes will include anthropometrical, functional, cardiometabolic, and musculoskeletal variables that provide complementary physiological context. Given the large number of endpoints and subgroup analyses, biopsy-derived outcomes (immunohistochemistry, electron microscopy, gene/protein expression, glycogen) and broad biochemical panels, as well as REDs/LEA screening outputs (e.g., questionnaires and energetic screening metrics), will be treated as exploratory, hypothesis-generating indicators, interpreted with caution given multiple comparisons.

Between-group differences in primary and secondary outcomes will be examined using factorial analysis of variance (ANOVA), with sex (female vs. male), age category, and training status (endurance-trained vs. sedentary) included as fixed factors. Where appropriate, interaction effects between sex, age category, and training status will be explored. HC status will be considered in stratified and sensitivity analyses in females; if group sizes permit, subgroup comparisons (naturally menstruating vs. HC) will be performed; otherwise, HC status will be included as a covariate. In the presence of significant main or interaction effects, post hoc comparisons with appropriate correction for multiple comparisons will be applied to control the family-wise error rate. Given the exploratory nature of some secondary outcomes, no formal adjustment for multiplicity is applied beyond post hoc family-wise error control. Accordingly, exploratory outcomes will be interpreted primarily as descriptive and hypothesis-generating, with emphasis on effect sizes and cross-domain consistency rather than on single-endpoint significance. For variables that violate assumptions of normality or homogeneity, non-parametric alternatives will be used as appropriate. In addition to *p*-values, effect sizes will be systematically reported to facilitate the interpretation of the magnitude and practical relevance of observed differences. Partial eta squared (η^2^_p_) will be reported for main and interaction effects derived from ANOVA models, while Cohen’s d will be calculated for pairwise comparisons. Effect sizes will be interpreted according to established conventions.

Associations between selected physiological, performance, and body composition variables will be explored using correlation analyses, with the choice of correlation coefficient determined by data distribution. Statistical significance will be set at *p* < 0.05.

## 3. Discussion

This study aims to examine how sex and ageing jointly influence physical fitness, physiological adaptations, endurance performance, and REDs risk in young and master endurance runners. A secondary aim is to determine whether long-term endurance running can preserve physiological function and mitigate age-related decline.

Extensive scientific evidence suggests that long-term endurance running in advanced age is effective in attenuating age-related physiological decline [11,72,73]. However, its effects differ between male and female runners due to various underlying, yet unclear, biological and physiological mechanisms. Therefore, a deeper understanding of the physiological changes associated with ageing and sex differences in endurance runners is crucial for their training optimisation and performance [1,2,74].

Current research highlights several critical gaps in knowledge, particularly regarding female endurance runners and the ageing process. The scientific evidence remains limited or inconsistent concerning long-term cardiovascular and musculoskeletal remodulations and adaptations in master female runners; endocrine system fluctuations associated with prolonged excessive endurance training; and the complex interactions between excessive endurance exercise, chronic low energy availability, and physiological maladaptation across the lifespan. These mechanisms warrant further investigation from physiological, health, and performance perspectives. Importantly, such research should not be limited only to female athletes, as growing evidence indicates REDs also affects male endurance runners, underscoring the need for sex-inclusive research approaches.

### 3.1. Expected Outcomes of the Study

It is expected that endurance-trained master athletes will demonstrate more favourable cardiorespiratory fitness and body composition profiles compared with age-matched sedentary controls. Differences between training status, age category, and sex are anticipated to emerge across selected physiological and cardiometabolic outcomes, reflecting long-term adaptations to endurance exercise and the ageing process.

Regarding REDs-related screening and LEA indicators, our working hypothesis is that master endurance runners may show equal or lower prevalence of “high-risk” REDs screening profiles than younger endurance runners, despite sustained training exposure. This expectation is based on emerging endurance-runner data indicating a lower proportion of “at-risk” screening classifications in older or “master” vs. younger female runners, alongside age-related differences in body-composition phenotype and reproductive and endocrine context [61]. At the same time, contemporary consensus statements emphasise that REDs/LEA remains possible at any age and can present with heterogeneous, partly subclinical manifestations; therefore, we also anticipate that master runners may still exhibit specific REDs-related indicators (e.g., low FFM, altered endocrine milieu, bone-related risk markers) even when global screening scores appear less severe [14,55,59,60,74,75,76,77,78].

### 3.2. Strengths of the Study

The main strength of this cross-sectional study is its comprehensive and multimodal assessment strategy. The comprehensive methodology enables a holistic linkage between physical performance, nutritional status, and overall health status across age and sex. The study further benefits from a robust design that includes young and master endurance runners alongside their age- and sex-matched inactive counterparts, allowing for precise evaluation of the effects of ageing, long-term endurance training, and sex differences. Moreover, the use of standardised screening tools for LEA strengthens the ability to detect REDs risk in a clinically relevant age and sex-specific manner in endurance runners.

While the protocol is intentionally comprehensive, the multimodal design is well suited to an exploratory cross-sectional study in older athletes and allows us to characterise ageing- and sex-related phenotypes across performance, energetics, endocrine/metabolic health, and muscle tissue structure. This approach is expected to generate mechanistic hypotheses and identify convergent marker patterns that can be tested in future longitudinal and interventional studies.

### 3.3. Limitations of the Study

On the other hand, this study also has several important limitations. An important limitation of this study is that its cross-sectional design limits the ability to capture long-term physiological and health changes in the subgroups of the endurance runners. This short observational time frame is only focused on a single one-week period, where all assessments of subjects’ habitual weeks will be conducted.

Selection bias should also be acknowledged, as the groups of master endurance runners will represent a highly specific, motivated, and generally health-conscious subgroup and, therefore, will not be representative of the broader ageing population. This “healthy survivor” profile may attenuate observed age-related declines and may also underestimate the prevalence or severity of adverse health indicators compared with less active older adults; consequently, the generalisability of our findings will be strongest for older endurance runners rather than for ageing adults overall.

Nevertheless, studying master endurance runners will remain methodologically valuable because this cohort provides a well-established human model of “successful/healthy ageing”, allowing for the investigation of age-related physiological decline under conditions of sustained high training exposure, thereby reducing confounding by the typical age-related reduction in physical activity [55,72,79]. Accordingly, our findings will be interpreted primarily within the context of healthy, high-functioning older endurance athletes, with the recognition that translation to the general ageing population may be limited.

A further limitation of the study relates to the indirect estimation of VO_2_max from maximal power output during cycle ergometry. While this approach will improve feasibility and safety, particularly in older participants, it may systematically underestimate aerobic capacity in endurance runners, who typically achieve higher VO_2_max during treadmill running than during cycling, reflecting exercise-modality specificity. Moreover, the magnitude of the treadmill–cycle difference may vary by training background, as well as by age and sex, due to differences in cycling familiarity and mechanical efficiency, which could influence between-group comparisons. Moreover, cycling efficiency and the translation of Wmax to VO_2_max may vary by age and sex, which could influence between-group comparisons [80]. To reduce confounding from sex- and age-related differences in body composition and to support mechanistic interpretation, VO_2_max will also be analysed after normalisation to FFM. Therefore, VO_2_max results will be interpreted cautiously, with emphasis on consistent patterns across complementary performance and physiological outcomes rather than on VO_2_max in isolation.

Additionally, this study design does not allow for longitudinal monitoring of training load, dietary patterns, hormonal fluctuations, or menstrual-cycle-related variability in female participants, which may influence energy availability, hormonal responses, and REDs manifestation over longer time scales. Additionally, the REDs screening tools used (LEAF-Q, LEAM-Q, and IOC REDs CAT2) rely only on self-reported symptoms. Thus, these tools may be vulnerable to recall bias; however, their use in combination with objective physiological and biochemical measures strengthens overall interpretability. Moreover, the REDs risk categories, which derive from questionnaires, must be interpreted with caution, complemented by other objective physiological and biochemical markers collected in this study and afterwards interpreted by a responsible medical specialist.

Nevertheless, the large number of assessed outcomes increases the likelihood that some findings will remain primarily descriptive; therefore, inference will be guided by the predefined outcome hierarchy and by triangulation across related measures, rather than by isolated statistically significant results.

## 4. Conclusions

This study protocol presents a comprehensive cross-sectional framework to investigate the combined effects of ageing, biological sex, and long-term endurance running on physiological performance, musculoskeletal and bone health, nutritional status, and the risk of REDs development. The protocol is designed to generate a multidimensional dataset across young adult and master endurance runners and their age-matched inactive counterparts. Importantly, this protocol has clear clinical and practical relevance, providing a structured framework for translational application in endurance athletes across the lifespan.

## Data Availability

The data will be available upon request from the authors of the article.

## Supplementary Materials

The following supporting information can be downloaded at https://www.mdpi.com/article/10.3390/sports14030121/s1: Figure S1: The cross-sectional study design.; Supplementary File S1: SPIRIT 2025 Checklist, Supplementary File S2: Eligibility Criteria for Study Participation.

## Abbreviations

ACSM	American College of Sports Medicine
ALM	Appendicular Lean Mass
ALMI	Appendicular Lean Mass Index
ANOVA	Analysis of Variance
AU	Arbitrary Units (Used for sRPE-Derived Internal Training Load)
BCA	Bicinchoninic Acid Assay
BM	Body Mass
BMC	Bone Mineral Content
BMD	Bone Mineral Density
BMI	Body Mass Index
CAT	(original) IOC REDs Clinical Assessment Tool
CAT2	IOC REDs Clinical Assessment Tool 2
CD31	Cluster of Differentiation 31 (Endothelial Marker)
CEUs	Calcium Entry Units
cDNA	Complementary DNA
COX-2	Cyclooxygenase-2
CR-10	Borg Category-Ratio 10 Scale
CRU	Calcium Releasing Units
DXA	Dual-Energy X-Ray Absorptiometry
EA	Energy Availability
EB	Energy Balance
ECG	Electrocardiography
ECL	Enhanced Chemiluminescence
eEB	Estimated Energy Balance
EEE	Exercise Energy Expenditure
EI	Energy Intake
EM	Electron Microscopy
eTEE	Estimated Total Energy Expenditure
FFM	Fat-Free Mass
FFMI	Fat-Free Mass Index
FoxO	Forkhead Box O (Transcription Factor Family; Atrophy-Related Marker)
HC	Hormonal Contraception
HOMA-IR	Homeostatic Model Assessment of Insulin Resistance
HRmax	Maximum Heart Rate
IL-1β	Interleukin 1 Beta
IL-6	Interleukin 6
IOC	International Olympic Committee
LBM	Lean Body Mass
LEA	Low Energy Availability
LEAF-Q	Low Energy Availability in Females Questionnaire
LEAM-Q	Low Energy Availability in Males Questionnaire
MET	Metabolic Equivalent of Task
METS	Metabolic Equivalents
MVC	Maximal Voluntary Contraction
MVPA	Moderate-To-Vigorous Physical Activity
MuRF-1	Muscle RING-Finger Protein-1 (Muscle Atrophy Marker)
MyoD	Myogenic Differentiation 1 (Myogenic Regulatory Factor)
NADPH	Nicotinamide Adenine Dinucleotide Phosphate
OCT	Optimal Cutting Temperature Compound
OXPHOS	Oxidative Phosphorylation (Protein Complex Marker Panel)
PAEE	Physical Activity Energy Expenditure
Pax7	Paired Box Protein 7 (Satellite Cell Marker)
PCR	Polymerase Chain Reaction
PT	Peak Torque
qPCR	Quantitative Polymerase Chain Reaction
REDs	Relative Energy Deficiency in Sport
RER	Respiratory Exchange Ratio
RMR	Resting Metabolic Rate
RNA	Ribonucleic Acid
RPLP0	Ribosomal Protein Lateral Stalk Subunit P0 (Reference Gene)
RTD	Rate of Torque Development
SD	Standard Deviation
SHBG	Sex Hormone-Binding Globulin
SPIRIT	Standard Protocol Items: Recommendations For Interventional Trials
sRPE	Session Rating of Perceived Exertion
SR	Sarcoplasmic Reticulum
SYBR	SYBR Green (Fluorescent DNA-Binding Dye Used in qPCR)
T_Low	Time Spent in Light-Intensity Activity
T_Mod	Time Spent in Moderate-Intensity Activity
T_Vig	Time Spent in Vigorous-Intensity Activity
TEE	Total Energy Expenditure
TEF	Thermic Effect of Food
TNFα	Tumour Necrosis Factor Alpha
TRI	TRI Reagent (RNA Isolation Reagent)
VE	Ventilation
VO_2_	Oxygen Uptake
VO_2_Max	Maximal Oxygen Uptake
VO_2_Peak,Abs	Absolute Peak Oxygen Uptake
VO_2_Peak,FFM	Peak Oxygen Uptake Normalised to Fat-Free Mass
Wmax	Maximal Power Output
YBT	Y-Balance Test
