# Supplementary material for: Ageing, Sex Differences, and REDs Risk in Endurance Runners: An Integrated Cross-Sectional Study Protocol
Source: Sports (Basel). 2026 Mar 19;14(3):121. doi: 10.3390/sports14030121 (PMC13030092; doi:10.3390/sports14030121)
Supplement: Supplementary file 1 [file sports-14-00121-s001.zip › Supplementary File S1.pdf]

SPIRIT 2025 Checklist

SPIRIT Item	Completed Information
1a	Title: Ageing, Sex Differences, and RED-S Risks in Endurance Runners – Cross-sectional Study Protocol.
1b	Structured summary including design, population, sites, outcomes, and registration (NCT05053282).
2	Protocol Version 1.0 (January 2025).
3a	**Ľudmila Oreská**[1,2]<br>• 1 Department of Biological and Medical Sciences, Faculty of Physical Education and Sports, Comenius University in Bratislava, 814 69 Bratislava, Slovakia<br>• 2 Department of Health Technologies, Faculty of Pharmacy, Comenius University in Bratislava, 832 32 Bratislava, Slovakia
3b	Sponsor: Comenius University Bratislava.
3c	Sponsor role: No involvement in design, data, or publication decisions.
3d	Committees: Not applicable (observational design).
4	Trial registration: ClinicalTrials.gov – NCT05053282.
5	Protocol and SAP accessible in journal and upon request.
6	Data sharing plan: De-identified datasets available upon request.
7a	Funding: APVV-21-0164; VEGA 0554/24; VEGA 1/0482/23.

7b	Conflicts of interest: None declared.
8	Dissemination: Peer-reviewed publication, conferences, feedback to participants.
9a	Scientific background: Ageing, sex differences, RED-S rationale.
9b	Comparator: Age- and sex-matched inactive controls.
10	Objectives: Primary and secondary aims clearly defined.
11	Patient/public involvement: None (laboratory-based).
12	Design: Cross-sectional observational study.
13	Setting: Comenius University labs; Sports Medicine Centre.
14a	Eligibility criteria for participants (inclusion & exclusion).
14b	Eligibility for personnel/sites: Not applicable.
15a	Interventions: Not applicable (observational).
15b	Criteria for modification: Not applicable.
15c	Adherence strategies: Not applicable.
15d	Concomitant care: Not applicable.
16	Outcomes: VO2peak, DXA, strength, RED-S tools, biomarkers, biopsies.
17	Harms monitoring: Minimal risk; biopsy/VO2 testing adverse events logged.
18	Timeline: Screening → monitoring → testing → biopsy.

19	Sample size determination using G*Power (112 participants).
20	Recruitment strategy: Clubs, university networks, senior groups.
21-24	Randomization/blinding: Not applicable.
25a	Data collection methods: VO2peak, DXA, dynamometry, biomarkers, IHC.
25b	Retention: High retention expected; no follow-up loss anticipated.
26	Data management: Encrypted storage, coded identifiers.
27a-d	Statistical methods: ANOVA, regression, missing data handling.
28a-b	Monitoring committees & interim analysis: Not applicable.
29	Monitoring: Internal oversight by PI.
30	Ethics approval: University Hospital Bratislava (No. 31/2020).
31	Protocol amendments: Reported to ethics board and registry.
32a	Consent: Obtained in person by trained researchers.
32b	Additional specimen consent: Included.
33	Confidentiality: Coded data, secure storage.
34	Post-trial care: Medical oversight; no long-term care required.
