# Supplementary material for: Ageing, Sex Differences, and REDs Risk in Endurance Runners: An Integrated Cross-Sectional Study Protocol
Source: Sports (Basel). 2026 Mar 19;14(3):121. doi: 10.3390/sports14030121 (PMC13030092; doi:10.3390/sports14030121)
Supplement: Supplementary file 1 [file sports-14-00121-s001.zip › Supplementary File S2.pdf]

Supplementary File S2. Eligibility Criteria for Study Participation

S2.1 Inclusion Criteria

Participants were eligible for the study if they met all of the following criteria:

1. **Physical Activity Profile**
   - **Highly active group:** Women aged 65–75 years who consistently performed **>300 minutes of endurance running training per week** over the last five years.
   - Endurance running, consistently maintained for at least three years, and compete at the national or international level
   - **Low activity group:** Women aged 65–75 years or adults aged 20–30 years performing **<30 minutes of physical activity per week** over the last five years.
2. **Age Requirements**
   - Older adult cohort: **65–75 years**
   - Young adult control group (if applicable to comparative analyses): **20–30 years**
3. **Language Proficiency**
   - Ability to **read and write in Slovak language**.
4. **Geographical Availability**
   - Residence within **a 1-hour travel distance** (by car or public transport) from the research facility.
5. **Anthropometric Requirements**
   - **Body Mass Index (BMI): 18.5–35.0 kg/m^2**
6. **Informed Consent**

- Written informed consent signed after receiving comprehensive written information on the study aims, procedures, measurements, associated risks, and participant responsibilities.

S2.2 Exclusion Criteria

Participants were excluded if they met any of the following conditions:

A. Medical and Health-Related Conditions

1. **Musculoskeletal limitations**
   - Disorders or injuries of the locomotor system, restricted mobility, or conditions impairing safe physical performance.
2. **Infections**
   - Acute or chronic infections precluding participation.
3. **Chronic diseases or clinical conditions** (diagnosed or medically treated), including:
   - Cardiovascular diseases (e.g., unstable angina, recent myocardial infarction <6 months, NYHA III–IV heart failure, severe arrhythmias, uncontrolled hypertension >160/95 mmHg)
   - Neurological disorders (e.g., epilepsy, recent stroke <6 months)
   - Pulmonary diseases (e.g., COPD, severe asthma, pulmonary/pleural/pericardial disease)
   - Metabolic and autoimmune diseases (e.g., insulin-dependent diabetes mellitus)
   - Active cancer or history of high-risk malignancies (e.g., prostate cancer)
   - Severe osteoporosis with unstable bone lesions or high fracture risk
4. **Nutritional disorders**
   - Diagnosed malnutrition, suspected malnutrition, or significant underweight.

B. Medication Use

5. Use of medications that interfere with biological analyses or bias outcomes, including:

- Glucocorticoids and other steroids
- Non-steroidal anti-inflammatory drugs (NSAIDs) within 24 h prior to any study visit
- Immunosuppressive therapies
- Antineoplastic (oncologic) treatment
- Hormonal replacement affecting metabolic parameters (e.g., insulin, testosterone)
- Beta-blockers
- Statins

C. Psychological and Cognitive Conditions

6. **Mental health or cognitive disorders**, including:
   - Severe anxiety or depressive states
   - Dementia
   - Alcoholism or substance abuse
   - Disorders affecting cooperation or comprehension of study procedures

D. Conditions Limiting Physical Performance

7. **Physical limitations preventing safe participation**, including:
   - Severe arthritis or chronic uncontrollable pain
   - Planned knee or hip arthroplasty
   - Pathological fractures (<6 months)
   - Amputations
   - Dependence on walkers or wheelchairs

E. Use of Substances Enhancing Physical Performance

8. **Current or past use of performance-enhancing substances**, doping agents, or other ergogenic drugs.

S2.3 Notes on Harmonization of Criteria Across Age Groups

To maintain methodological consistency across older and younger cohorts, inclusion and exclusion criteria were aligned wherever possible. Medication- and condition-based exclusions were applied uniformly to avoid confounding physiological outcomes, particularly in analyses involving biological samples (muscle biopsies, blood biomarkers, hormonal profiles).

S2.4 Summary Statement

These criteria ensured the recruitment of a well-defined population representing either long-term endurance-trained older adults or low-activity controls, free from comorbidities or treatments that could alter physiological, metabolic, or molecular responses examined within the study.
