# Supplementary figures and images for: Ageing, Sex Differences, and REDs Risk in Endurance Runners: An Integrated Cross-Sectional Study Protocol
Source: Sports (Basel). 2026 Mar 19;14(3):121. doi: 10.3390/sports14030121 (PMC13030092; doi:10.3390/sports14030121)

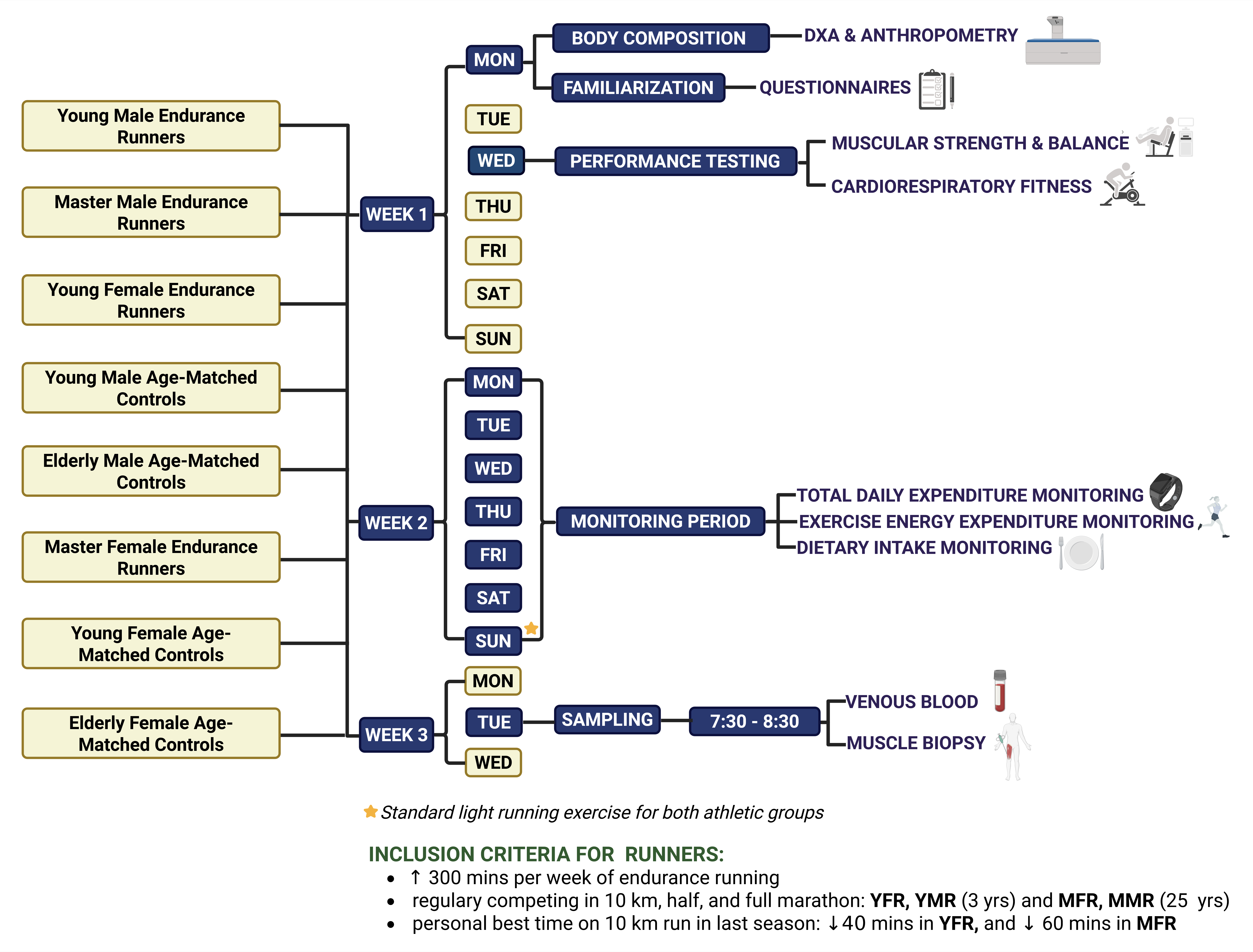

Supplement: Supplementary file 1 [file sports-14-00121-s001.zip › Figure S1.jpeg]
